# Mesenchymal Stem Cell Migration and Tissue Repair

**DOI:** 10.3390/cells8080784

**Published:** 2019-07-28

**Authors:** Xiaorong Fu, Ge Liu, Alexander Halim, Yang Ju, Qing Luo, Guanbin Song

**Affiliations:** 1 College of Bioengineering, Chongqing University, Key Laboratory of Biorheological Science and Technology, Ministry of Education, Chongqing 400030, China; 2 Department of Mechanical Science and Engineering, Nagoya University, Nagoya 464-8603, Japan

**Keywords:** mesenchymal stem cells, migration, differentiation, paracrine, tissue repair, mechanochemical regulation

## Abstract

Mesenchymal stem cells (MSCs) are multilineage cells with the ability to self-renew and differentiate into a variety of cell types, which play key roles in tissue healing and regenerative medicine. Bone marrow-derived mesenchymal stem cells (BMSCs) are the most frequently used stem cells in cell therapy and tissue engineering. However, it is prerequisite for BMSCs to mobilize from bone marrow and migrate into injured tissues during the healing process, through peripheral circulation. The migration of BMSCs is regulated by mechanical and chemical factors in this trafficking process. In this paper, we review the effects of several main regulatory factors on BMSC migration and its underlying mechanism; discuss two critical roles of BMSCs—namely, directed differentiation and the paracrine function—in tissue repair; and provide insight into the relationship between BMSC migration and tissue repair, which may provide a better guide for clinical applications in tissue repair through the efficient regulation of BMSC migration.

## 1. Introduction

Mesenchymal stem cells (MSCs) represent an important source for cell therapy in regenerative medicine. MSCs have shown promising results for repairing damaged tissues in various degenerative diseases, both in animal models and in human clinical trials [1,2,3,4,5,6]. MSCs have a homing ability, meaning that they can migrate into injured sites, and they possess the capacity to differentiate into local components of injured sites and the ability to secrete chemokines, cytokines, and growth factors that help in tissue regeneration [7,8,9,10]. Bone marrow-derived mesenchymal stem cells (BMSCs) are non-hematopoietic stem cells present in the bone marrow and have multipotential differentiation potential [11,12].

BMSCs are the most used stem cells in cell therapy and tissue repair. In response to injury signals, BMSCs can potentially move from their niche into the peripheral circulation and pass through vessel walls to reach target tissues [13,14]. The efficacy of BMSCs in cell therapy depends on their homing ability and engraftment into the injury site for a long time [15]. However, the trafficking of BMSCs from their niche to target tissues is a complex process. This delivery process is affected by both chemical factors (such as chemokines, cytokines, growth factors) and mechanical factors (such as hemodynamic forces applied to the vessel walls in the forms of shear stress, vascular cyclic stretching, and extracellular matrix (ECM) stiffness). At present, some methods for evaluating cell migration have been developed. The commonly used methods to study BMSC migration in vitro are transwell and scratch wound assays [11,15]. Meanwhile, the tracing of transplanted BMSC migration in vivo is usually achieved by labelling BMSCs with fluorescence or luciferase, and then tracking them in vivo through a bioimaging system [2,5,9].

Researchers have conducted in-depth research on the factors that affect the homing mechanism of BMSCs and their characteristics of paracrine activity in tissue healing and differentiation into local parts of damaged sites. In this paper, we review the current understanding of BMSC homing and migration, with a focus on the chemical and mechanical factors that regulate BMSC migration and homing, hoping to provide assistance for clinical applications and the treatment of degenerative diseases.

## 2. Chemical Factors Regulating BMSC Migration

The factors involved in BMSC migration, homing, and functionality have been widely investigated. Studies have shown that the delivery of BMSCs to injured tissue sites is regulated by multiple chemical factors, for example, chemokines, cytokines, and growth factors (Table 1).

### 2.1. SDF-1/CXCR4 Axis

It has been extensively demonstrated that the stromal derived factor-1 (SDF-1)/CXC chemokine receptor 4 (CXCR4) axis is critical for BMSC homing. An in vitro study revealed that when the SDF-1 concentration was lower than 100 ng/mL, the number of migrated BMSCs increased as the concentration of SDF-1 increased. However, when the concentration exceeded 100 ng/mL, the number of migrated cells decreased as the concentration of SDF-1 increased [35]. A number of studies have shown that the SDF-1/CXCR4 axis is critical for inducing BMSC homing to the site of injury and for BMSC participation in the regeneration of tissue. For example, in vitro and in vivo research showed that SDF-1/CXCR4 played an important role in BMSC recruitment to injured liver [16]. Deng et al. showed that the mRNA and protein expressions of CXCR4 gradually increased as SDF-1 concentration increased, and transplanting BMSCs with SDF-1-induced CXCR4 expression can promote the repair of traumatic brain injury [17]. SDF-1 pretreatment increased the migration of Wharton’s jelly-derived mesenchymal stem cells (WJ-MSCs) and embryonic stem cells (ESCs) and enhanced skeletal muscle regeneration [18]. In addition, some studies have confirmed that the expression level of SDF-1 is significantly increased after tissue injury, and the recruitment of BMSCs expressing CXCR4 toward the SDF-1 gradient plays a crucial role in cardiac recovery [16,36,37].

Many studies have designed transfection or transduction experiments in which CXCR4 expression plasmids are introduced into BMSCs in a non-viral or viral manner. CXCR4 overexpression in BMSCs improved the homing of BMSC to bone marrow after intracardiac injection in a non-obese diabetic/severe combined immunodeficiency (NOD/SCID) model [38]. The overexpression of CXCR4 in MSCs enhanced chemotaxis toward SDF-1, and homing toward bone marrow was also found after CXCR4-overexpressing MSCs were transplanted by intramedullary and tail vein injections, respectively [39]. Cheng et al. also showed that overexpression of surface CXCR4 increased the engraftment of BMSCs in infarcted myocardium and improved cardiac performance [40].

These results suggest that the SDF-1/CXCR4 axis plays an important role in the regulation of the migration of BMSCs. Increasing CXCR4 expression might be a potential strategy to improve the migration ability of BMSCs and accelerate the tissue repairing efficiency.

### 2.2. Osteopontin (OPN)

OPN is an O-glycosyl phosphate protein that is synthesized in various tissues, it is also a cytokine upregulated in response to an injury and inflammation in the heart, kidney, lung, bone, and other tissues. Increase in OPN expression was related to increase in cell migration and survival ability in various cells [41,42], and in particular was able to increase the migration of MSCs. Studies showed that the migration of BMSCs to OPN occurred in a concentration-dependent manner [19,20]. Further mechanism study revealed that OPN increased integrin β1 expression in BMSCs and promoted BMSC migration through the ligation to integrin β1 [19].

The ability of the cell body to dynamically reshape itself is important for migration behavior because of the reduced physical challenges that occur within the tissue. How a cell remodels its shape is related to its cell stiffness, and cell stiffness is determined by the structure of the cytoskeleton [43]. The nucleus, on the other hand, is stiffer than the surrounding cytoplasm, and its size and stiffness are a major barrier to cell migration through narrow openings [44]. Our previous study showed that in vitro treatment with OPN reduced the number of organized actin cytoskeletons through FAK-ERK1/2 pathways, which resulted in enhanced BMSC migration due to cell stiffness reduction observed by atomic force microscopy [21]. Further investigation into the changes in the mechanical properties of the treated OPN in the nucleus revealed that OPN could reduce the nuclear stiffness of BMSCs and reduce the expression of lamin A/C, which is the main factor of the nuclear stiffness, via FAK-ERK1/2 pathways to increase BMSC migration [22]. Interestingly, it was also found that the cytoskeleton plays an important role in the OPN-increased BMSC migration by controlling the morphology and stiffness of the nucleus through the SUN1 protein, a key component of the nuclear cytoskeleton and cytoskeletal linker [23]. The chromatin organization was altered by the application of OPN via the ERK1/2 signaling pathway, which also contributed to BMSC migration [24].

### 2.3. Growth Factors

Growth factors are a class of polypeptides that regulate cell migration, proliferation, differentiation, and extracellular matrix synthesis. Studies have found that growth factors play an important role in the migration of MSCs. Currently, basic fibroblast growth factor (bFGF), vascular endothelial growth factor (VEGF), hepatocyte growth factor (HGF), insulin-like growth factor-1 (IGF-1), platelet-derived growth factor (PDGF), and transforming growth factor β1 (TGF-β1) are commonly used in tissue repair. A number of studies have shown that these growth factors are critical for inducing MSC homing to the site of injury and for the involvement of MSCs in tissue regeneration.

#### 2.3.1. bFGF

bFGF was reported to be a potent mitogen that can stimulate migration in various cell types [45,46] and, in particular, can increase the migration of BMSCs and play an important role in BMSC homing to injured sites [25]. A bFGF gradient experiment showed that a low concentration of bFGF promotes BMSC migration, while a high concentration of bFGF inhibits BMSC migration, and this contradictory effect of bFGF provides a possibility for BMSC directional routing [26]. Furthermore, bFGF could increase the recovery of damaged sites. For example, platelet-derived bFGF recruits human BMSCs to the endothelium and induces BMSCs to participate in the restoration of vascular integrity after endothelial injury [47]. Retrograde infusion of BMSCs with concomitant bFGF results in enhanced cardiac function recovery after myocardial infarction [25]. Further studies revealed that bFGF increases BMSC migration by up-expression of αVβ3 integrin and activation of MEK/ERK pathways and phosphatidylinositol 3-kinase (PI3K)–dependent Akt (PI3K/AKT) pathways [26].

#### 2.3.2. VEGF

VEGF and its receptor VEGFR are crucial regulators of the growth, development, and migration of several types of cells [48]. The most abundant and active member of the endothelial growth factor family is VEGF-A [49,50]. Studies showed that VEGF-A could stimulate platelet-derived growth factor receptors (PDGFRs), thereby regulating human BMSC migration and proliferation [27]. Overexpressing VEGF in human BMSCs stimulates SDF-1α expression in infarcted hearts and results in massive mobilization and homing of BMSCs and cardiac stem cells, which are beneficial to a reduction in infarct size [48]. These results provide important new insights into how VEGF regulates BMSC recruitment during tissue regeneration and disease.

#### 2.3.3. HGF

HGF is a pleiotropic growth factor of mesenchymal origin, which can promote the motility, proliferation, migration, and survival of a wide spectrum of cells [28,51]. The expression of c-met, the cognate receptors of HGF, in human BMSCs are strongly attracted by HGF concentration gradients, resulting in increased trafficking of MSCs in vitro [28]. Some key signaling molecules are involved in HGF-induced MSC migration. For example, a study showed that HGF increased rat BMSC migration via increasing the expression of microRNA-221 and microRNA-26b through the activation of the Akt and FAK pathways [52]. The exposure of mouse BMSCs to HGF increased migration via the PI3K-dependent pathway [28].

#### 2.3.4. IGF-1

IGF-1 can induce the migration and proliferation of multiple cell types [29,53]. Studies showed that IGF-1 could regulate BMSC migration and have implications for the development of novel stem cell therapeutic strategies [7,30]. Overexpressing IGF-1 in rat BMSCs improved survival and engraftment in the infarcted heart and promoted stem cell recruitment through paracrine release of SDF-1 [7]. Preconditioning of mouse BMSCs with IGF-1 before infusion improved cell migration capacity and restored normal renal function after acute kidney injury [30]. An exploration of the underlying mechanism found that IGF-1 increased expression levels of the SDF-1 receptor CXCR4 in rat BMSCs, which markedly increased the migratory response of BMSCs to SDF-1 via the PI3K pathway [29]. These results demonstrate that promoting the migration of BMSCs by IGF-1 could increase their therapeutic potential and indicate a new therapeutic paradigm for organ repair.

#### 2.3.5. PDGF

PDGF is a polypeptide dimer released by platelets during degranulation [8]. The polypeptides are connected by disulfide bonds and exist as isoforms A, B, C and D [54]. Human platelets contain PDGF-AB, PDGF-BB and PDGF-CC, which bind to both PDGF receptors (PDGFR-α and PDGFR-β) [55,56]. One study showed that human BMSCs exhibit significant chemotaxis responses to several factors, including fetal bovine serum, PDGF, VEGF, and IGF-1, while PDGF is the most potent factor that increases BMSC migration [31], suggesting that PDGF may be a most potent growth factor in BMSC recruitment and tissue repair. Local resting resident fibroblasts are activated after injury and play a critical role in recruiting BMSCs. PDGF-BB-activated fibroblasts caused significant increases in mouse BMSC migration in an in vitro wound healing assay [32].

PDGFR also plays an important role in inducing BMSC migration. When GFP-expressing human placenta MSCs were topically applied into excisional wounds in mice, PDGFR-β^+^ MSCs (MSCs expressing PDGF receptor, PDGFR-β) actively incorporated into the wound tissue, resulting in enhanced engraftment and accelerated wound closure compared to PDGFR-β^−^ MSCs (MSCs not expressing PDGFR-β), indicating that PDGFR-β identifies MSCs with enhanced chemotactic migration to the wound injury [57]. In an orthotopic transplantation model of human colon cancer, a blockade of PDGFR signaling inhibited the migration and survival of MSCs in the tumor microenvironment, hence, it inhibited the progressive growth of colon cancer [58]. Neonatal lung MSCs from infants who develop bronchopulmonary dysplasia showed lower PDGFR-α and PDGFR-β mRNA and protein expression levels and decreased migration to PDGF treatment, demonstrating that defective PDGFR signaling affects MSC migration [59]. These findings provide new insights into the molecular mechanisms of PDGF-induced migration of MSCs, which may be relevant to control MSC function and tissue remodeling after injury or some diseases.

#### 2.3.6. TGF-β1

As a secretory polypeptide signal molecule, TGF-β1 has extensive biological activity. Study showed that the TGF-β1 secretory level increased in injured parts, and it is involved in the repair of damaged sites [9,60]. The in vivo experiment showed that the expression of TGF-β1 increased in the ischemia/reperfusion injury of mice myocardial tissue, which induced the homing of BMSCs for the repair of myocardial injury, by regulating the expression of CXCR4 [9]. In mice models of asthma induced by cockroach allergen extract (CRE), there were higher levels of active TGF-β1 in the lungs of the CRE-treated mice observed, which increased the recruitment of BMSCs after systemic injection of GFP^+^ BMSCs [61]. Pretreatment of murine BMSCs with TGF-β1, which is highly expressed in injury sites, improved wound closure in a syngeneic murine wound model [33]. Study showed that the N-cadherin, PI3K/Akt, ERK1/2, FAK, and p38 signal pathways were involved in the migration of human BMSCs in response to TGF-β1 [34]. These results demonstrate that for injury sites, secreting TGF-β1 is a crucial way to recruit BMSCs, and that pre-conditioning with TGF-β1 may be an efficient way to increase BMSC homing and migration.

Obviously, from what is described above, it is shown that the chemical factors affecting BMSC migration are complex. In this section, we only focused on several principal chemical factors. Actually, more chemical factors were also reported to be involved in mediating BMSC migration, such as chemokine (C-X-C motif) ligand 7 (CXCL7) [62], Leu-Leu 37 (LL-37) [63], fms-like tyrosine kinase 3 (Flt3) [64], and stem cell factor (SCF) [65]. Moreover, the bone remodeling process is also one of the most prominent physiological mechanisms that induces MSC migration, which is critical for bone fracture healing [66]. Hence, a comprehensive understanding of the chemical factors affecting the migration of BMSC is worthwhile for future study.

## 3. Mechanical Factors Regulating BMSC Migration

The microenvironment in which BMSCs live plays an important role in their migration. Besides chemical factors, the migration of BMSCs is also affected by mechanical factors such as mechanical strain, shear stress, matrix stiffness, and microgravity (Table 2).

### 3.1. Mechanical Strain

During the process of migration to the sites of injured tissue through the peripheral blood circulation, BMSCs adhere to the blood vessel wall and are subjected to hemodynamic forces applied to the vessel walls, in the forms of cyclic mechanical strain and blood shear stress. Studies showed that the migration of BMSCs was affected by mechanical strain. For example, in an in vitro study, mechanical strain (5%, 6 h) increased human BMSC migration. Researchers transplanted BMSCs into an in vivo animal model of skin tissue expansion and tracked BMSC migration and confirmed the contribution of migrating BMSCs to skin regeneration in the presence of mechanical stretching, through feedback of the expression between MMP2 and the SDF-1/CXCR4 axis [67]. Our previous study showed that cyclic mechanical stretching (10%, 8 h) promotes BMSC migration via the FAK-ERK1/2 signaling pathway [68] but decreases BMSC invasion through MT1-MMP downregulation via the PI3K/Akt signaling pathway [69]. In vivo and in vitro results showed that mechanical stretching (10%, 12 h) can upregulate BMSC migration and recruit circulating BMSCs through the SDF-1α/CXCR4 pathway to increase skin regeneration [70].

### 3.2. Shear Stress

Another kind of hemodynamic force applied to the vessel walls is shear stress. To date, studies of shear stress on MSC migration remain limited in number. Our previous studies showed that, detected by scratch wound assay, the wound closure rate of human MSCs was significantly faster than that of static cultured MSCs, under shear stress of 0.2 Pa, by activating JNK and p38 MAPK pathways, while shear stress >2 Pa significantly inhibited the migration of human MSCs via inhibiting the JNK and p38 MAPK pathways [71]. Further study found that shear stress (0.2 Pa) upregulated the secretion of SDF-1, which stimulated its receptor CXCR4 expression in human MSC migration via the JNK and p38 MAPK pathways [71]. In addition, our previous study indicated that shear stress (0.2 Pa) induced the migration rate of human MSCs along the downstream edge of the wound significantly faster than it did for cells along the upstream edge of the wound, and the ERK1/2 pathway was phosphorylated earlier in the downstream than in the upstream MSCs [72].

### 3.3. Matrix Stiffness

MSCs are surrounded by an extracellular matrix (ECM) that transmits complex biochemical and biophysical signals [73,74]. The elastic modulus of the ECM is an example of a biophysical cue. MSCs perceive this modulus of elasticity by deforming their surroundings through the forces that they produce, commonly referred to as “stiffness” and measured in pascals or Pa [73]. The physical signals provided to MSCs play an important role in regulating the behavior of the MSCs. Many studies have focused on the effect of matrix stiffness on the migration of MSCs. Human BMSCs migrated from a soft matrix (1 kPa) to a stiff matrix (34 kPa) by polarizing the cytoskeleton function and phosphorylated myosin-II heavy chain [74], suggesting that polarization is a highly regulated guidance for mechanically sensitive migration of MSCs. The microtubule organizing center (MTOC) frequently polarizes to a position in front of the nucleus during cell migration, and ECM stiffness influences the position of the MTOC in human MSCs by polarizing it in front of the nucleus only when the matrix is sufficiently stiff (≥5–6 kPa) during cell migration [75]. One experiment involved constructing in vitro stiffness gradients of different substrates, natural tissue stiffness variations (1 Pa/µm), pathological conditions (10 Pa/µm), and tissue interfaces that presented step changes in stiffness (>100 Pa/µm), to simulate the stiffness environment of different substrates in vivo, which were within an identical range of stiffness of relevance to physiological soft tissue (1–12 kPa). The results of this experiment showed that human MSCs migrated to stiffer portions of the substrates via functional actin cytoskeleton, and the assembled microtubule network was necessary for directed migration of MSCs [73]. It is important to note that MSCs also migrate in response to a soft matrix. Amniotic fluid-derived stem cells (AFSCs) cultured on softer substrates (2 kPa) secreted more autocrine cytokines, which increased AFSC migration compared to cells cultured on plastics (~100,000 kPa) by transwell assay [76]. A study involving epidermal growth factor-induced chemotaxis of human MSCs, through polydimethylsiloxane microchannels with varying substrate stiffness, showed that under an identical chemokine gradient, human MSCs migrated fastest on 3 kPa soft substrates associated with the formation of smaller adhesions when compared to stiffer substrates (30 kPa and 600 kPa) [77].

### 3.4. Microgravity

Normal gravity (1 g) is very important to maintain the normal functions of the body [78]. The microgravity environment during spaceflight induces related health problems, such as bone loss in weight-bearing bones [79]. Studies have shown that simulated microgravity conditions can inhibit the proliferation and differentiation of MSCs [80,81]. The migration of MSCs in microgravity and in normal gravity differs significantly. Our study showed that stimulated microgravity (rotated at 10 rpm, approximately 1 × 10^−3^ g) inhibited the migration of rat BMSCs via reorganizing F-actin and increasing cell stiffness [82]. With the culture of bone marrow hematopoietic stem cells (HSCs) in a modeled microgravity environment (rotated at 10 to 12 rpm, approximately 1 × 10^−3^ g to 1.2 × 10^−3^ g) for 2 to 3 days, the migration of HSCs was inhibited through a significant reduction of SDF-1α, which correlated with a decreased expression of F-actin [83]. At present, due to the limitation of experimental conditions, research on microgravity-affected MSC migration and mechanisms has not been widely carried out. However, considering the important role of MSCs in tissue repair and the health problems of pilots in the space environment [79], this is a very worthy field of further study.

**Table 2:** Effect of mechanical factors on BMSC migration

Mechanical Regime	Cell Migration	Outcomes	References
Mechanical stretch (5%, 6 h)	↑	Enhanced homing and transdifferentiation of BMSCs under mechanical stretch in the expanded skin, and BMSCs were recruited to sites where SDF-1αwas most highly expressed.	[67]
Mechanical stretch (10%, 8 h)	↑	Promoted BMSC migration via FAK and ERK1/2 signals.	[68]
Mechanical stretch (10%, 12 h)	↑	In vivo and in vitro results showed that mechanical stretch can upregulate SDF-1α in skin and recruit circulating BMSCs through the SDF-1α/CXCR4 pathway.	[70]
Shear stress (0.2 Pa/>2 Pa)	↓↑	High shear stress (>2 Pa) hindered human BMSC migration, whereas lower shear stress (0.2 Pa) induced cell migration.	[71]
Shear stress (0.2 Pa)	↑	The SDF-1/CXCR4 axis mediated low-shear-stress-induced human BMSC migration through the JNK and p38 MAPK pathways.	[72]
Matrix stiffness (1 kPa, 2.3 h; 34 kPa, 6.3 h)	↑	BMSCs migrated from the soft matrix to the stiff matrix by polarizing the cytoskeleton function and the phosphorylated myosin-II heavy chain.	[74]
Matrix stiffness (≥5-6 kPa, 2 h)	↑	Extracellular matrix (ECM) stiffness influenced the position of the microtubule organizing center (MTOC) in MSCs by polarizing it in front of the nucleus only when the matrix was sufficiently stiff, which increased MSC migration.	[75]
Matrix stiffness (1 to 12 kPa, 3 days)	↑	Human MSCs migrated to stiffer portions of the substrates by increasing the assembled microtubule network.	[73]
Matrix stiffness (2 kPa, 4 h)	↑	AFSCs cultured on softer substrates secreted more autocrine cytokines, which increased AFSC migration.	[76]
Microgravity (rotated at 10 rpm, approximately 1 × 10^−3^ g; 24 h)	↓	The migration of BMSCs was inhibited by simulated microgravity via reorganizing F-actin and increasing cell stiffness.	[82]
Microgravity (rotated at 10 to 12 rpm, approximately 1 × 10^–3^ g to 1.2×10^–3^ g; 2 to 3 days)	↓	The culture of HSCs in a microgravity environment inhibited the migration of HSCs by a significant reduction of SDF-1α-directed migration, which correlated with a decreased expression of F-actin.	[83]

## 4. Mechanisms of BMSCs in Tissue Repair

After mobilization and migration into injured tissues, BMSCs will perform functions and promote wound healing of damaged tissues and diseases. Studies showed that, during this repairing process, recruited BMSCs secrete chemical factors—such as chemokines, cytokines, and growth factors—which are known as paracrine and are necessary to promote tissue repair/regeneration [2,84] and/or differentiation into the injured tissue [28,84]. Of note, the paracrine function has recently received more credit as compared to direct MSC differentiation. In this section, we discuss the two critical roles of BMSCs in tissue repair.

### 4.1. BMSC Paracrine Factors and Tissue Repair

#### 4.1.1. Paracrine Factors of Transplanting BMSCs

Currently, a number of studies have shown that transplanting MSCs in vivo can increase injured site closure by secreting paracrine factors, which are beneficial to the healing of damaged tissue (Table 3). For example, after myocardial infarction, rat BMSCs secrete paracrine factors (TGF-β, FGF-2, angiopoietin-2, VEGF-1) to trigger angiogenic and migratory effects at the site of the infarct to promote myocardial healing and improve the cardiac function [1]. In a NOD/SCID mouse model, the transplanted BMSCs secreted nerve growth factor (NGF), HGF, and anti-inflammatory molecules (IL-10, IL1-RA), which contribute to the prevention of apoptosis and increase cell proliferation in the damaged liver [84]. Immunoblotting analysis found that the expression of neovascularization-related genes, such as *TGF-β1* and *VEGF*, increased in a BMSC-treated mouse burn injury model [2]. In a mouse acute kidney injury model, mouse BMSCs exert beneficial effects on tubular cell repair in acute kidney injury by producing the mitogenic and pro-survival factor IGF-1 [85]. The secretion of four major factors (angiogenin, IL-8, MCP-1, and VEGF) by WJ-MSCs increased vascular regenerative efficacy in a mouse hind limb ischemia model [4]. In a rat middle cerebral artery occlusion ischemia model, Wakabayashi et al. revealed that intravenously transplanted BMSCs induced functional improvement, reduced infarct volume, and enhanced neuroprotection in ischemic rats, possibly by providing IGF-1 and inducing VEGF, EGF, and bFGF neurotrophic factors in the host brain [86]. Human BMSC-generated TGF-β was shown to be a key player in suppressing immune propagation by decreased ischemic damage-induced MCP-1 expression in a rat stroke model [87]. Rat BMSCs’ overexpression of the *SDF-1* gene enhances the secretion of biologically active SDF-1, VEGF, HGF, and IL-6, which promotes the activity of dermal fibroblasts and keratinocytes to promote re-epithelialization and angiogenesis and, consequently, facilitates wound healing of rat skin wounds [5].

#### 4.1.2. Conditioned Medium from BMSCs for Tissue Repair

MSCs have become an attractive cell source and are widely employed in the development of a variety of regenerative medicine and tissue repair strategies. However, it has also been reported that MSC transplantations may result in some dangerous disadvantages, such as the formation of teratomas by MSCs. To avoid this possible safety concern, recent attention has been focused on using BMSC conditioned medium (BMSC-CM), containing internal cytokines/mediators secreted by BMSCs, to develop a cell-free therapeutic approach in stem cell therapy (Table 4). Kawai et al. clarified that the use of BMSC-CM is an alternative therapy for periodontal tissue regeneration, because several cytokines (IGF-1, VEGF, TGF-β1, and HGF) were included in BMSC-CM that contribute to wound healing and angiogenesis [3]. The intramuscular injection of conditioned medium (involving IL-6 and IL-8) derived from TNF-α-treated human BMSCs into a rat hindlimb ischemia model stimulated angiogenesis and tissue repair [88]. It was found that conditioned media from human umbilical cord blood-derived mesenchymal stem cells (UC-MSC-CM) contain many skin rejuvenation-associated paracrine factors—such as epithelial growth factor (EGF), bFGF, PDGF, HGF, collagen type 1, and, especially, one of the rejuvenation factors, namely, growth differentiation factor-11 (GDF-11)—which can induce skin wound healing by increasing the growth and ECM production of human dermal fibroblasts [89]. Fibroblasts are the primary cells involved in wound repair. Under dermal UC-MSC-CM stimulation, the characteristics of adult fibroblasts altered to produce high ratios of collagen types III and I and a high MMPs/TIMPs ratio, suggesting that UC-MSC-CM may be a feasible strategy to promote cutaneous repair and a potential way to heal damaged sites [90].

### 4.2. Directed Differentiation of BMSCs and Tissue Repair

Apart from the paracrine mechanism, another key mechanism of MSCs in tissue repair is their directed differentiation into damaged tissue. Numerous studies have confirmed that the directed differentiation of BMSCs into components of damaged tissue is essential for injury healing.

Treatment with HGF on mouse BMSCs induced the expression of cardiac-specific markers, with the concomitant loss of the stem cell markers nucleostemin, c-kit, and CD105 [28]. Rat BMSCs in varying neural differentiation states display different chemotactic responses to HGF, thereby shedding light on the optimization of the therapeutic potential of MSCs to be employed for neural regeneration after injury [91]. Intravenous transplantation of BMSCs into a rat tissue expansion model was able to effectively promote expanded skin regeneration via differentiating into CD31^+^ endothelial cells under mechanical stretch [92]. The infused human BMSCs in a NOD/SCID mouse model expressed cytokeratin CK18, CK19, and AFP, indicating a hepatocyte differentiation against radiated liver injuries [85].

Study showed that the high mobility group box 1 (HMGB1)/receptor for advanced glycation end product (RAGE) axis is involved in regulating the differentiation of MSCs. HMGB1 stimulated rat BMSCs to migrate and differentiate to endothelial cells via RAGE signaling [93]. Rat BMSCs can differentiate into endothelial lineage via Akt and NF-κB pathways, in response to a hypoxic microenvironment [11]. RhoA/ROCK, cytoskeletal organization, and FAK are essential for mechanical stretch-induced tenogenic differentiation of BMSCs characterized by the upregulation of tendon-related marker gene expression [12]. These findings suggest that related signaling pathways play a crucial mediating role in MSC differentiation induced by chemical factors, mechanical factors and coupling of mechanochemical factors. Unfortunately, very little is known about these molecular mechanisms.

## 5. Conclusions and Perspectives

**Figure 1:**
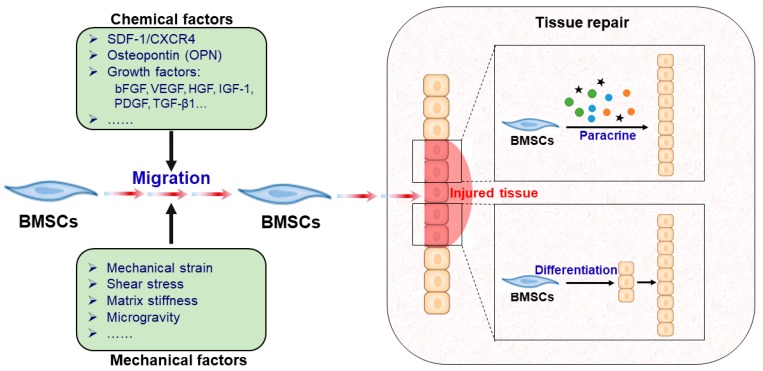
Proposed schematic diagram of the relationship between BMSC migration and tissue repair. Once BMSCs sense the injury signal released from damaged tissue, they migrate from bone marrow into the injured tissue through peripheral circulation, which is regulated by multiple mechanical and chemical factors. After reaching the damaged tissue site, BMSCs perform wound healing of damaged tissues through two key roles: the paracrine mechanism and/or directed differentiation.

In summary, it is well confirmed that BMSCs play important roles in tissue healing and regenerative medicine because of their self-renewal, migration, and pluripotency. A proposed schematic diagram of the relationship of BMSC migration and tissue repair and its involved mechanisms is shown in Figure 1. After sensing the injury signal released from damaged tissues, BMSCs can mobilize from bone marrow and migrate into injured tissues through peripheral circulation; this trafficking process is regulated by multiple mechanical and chemical factors. Subsequently, BMSCs reach the damaged tissue site and perform wound healing of damaged tissues through two key mechanisms, i.e., paracrine and/or directed differentiation.

In the past dozen years, a wealth of experimental evidence demonstrates the crucial repairing action of BMSCs in damaged tissue and disease. For example, it was found that the combination of MSC transplantation and physical exercise was more effective for the treatment of Parkinson’s disease in a rat model [94]. Our understanding of the key roles of BMSCs in tissue repair has largely increased. However, there are still many questions in this fields to be answered: (i) Although a wide range of mechanical and chemical factors affecting BMSC migration have been studied, most of these results are acquired from single factor experiments, at the cellular level, in vitro [77,95]. Actually, BMSC migration is influenced by multiple mechanical and chemical factors and their synergistic effect in vivo. This synergistic effect by multiple factors on BMSC migration in physiological conditions has not yet been fully investigated. (ii) Several reports demonstrate that BMSC migration and differentiation respond to gravity, and microgravity or zero gravity significantly affect the migration and differentiation of BMSCs, which suggests that an interesting and important research field is that of MSCs and tissue repair in space. Despite the many difficulties in this field due to the limited opportunity, it would be very worthwhile to explore the effect of the unique environment of space on BMSC migration and differentiation, which will provide a basic understanding for wound healing and pathophysiological alterations for astronauts in space flight. (iii) At present, a great variety of factors affecting BMSC migration and differentiation have been identified; however, the detailed mechanism involved in those regulation and control processes is not yet fully understood. Answers to these questions would advance our full understanding of the mechanochemical factors and the molecular basis affecting MSC migration and differentiation, as well as provide us with valuable information for further studies of the efficient regulation of MSCs’ biological behaviors in tissue repair, in basic research and clinical application.

## Figures and Tables

**Table 1:** Effects of chemical factors on bone marrow-derived mesenchymal stem cell (BMSC) migration

Chemical Factor	Concentration	Cell Migration	Outcomes	References
Stromal derived factor-1(SDF-1)	50 ng/mL, 100 ng/mL	↑	SDF-1 increased BMSC recruitment to injured liver and promoted the repair of injured liver.	[16]
SDF-1	100 ng/mL	↑	SDF-1 increased BMSCs with CXCR4 expression and promoted the repair of traumatic brain injury.	[17]
SDF-1	10 ng/mL	↑	SDF-1 increased stem cell recruitment, and the pretreatment of stem cells (Wharton’s jelly-derived mesenchymal stem cells (WJ-MSCs), embryonic stem cells (ESCs)) enhanced skeletal muscle regeneration.	[18]
Osteopontin (OPN)	1 μg/mL	↑	Increased integrin β1 expression in BMSCs and promoted BMSC migration through the ligation to integrin β1.	[19]
OPN	10 μg/mL, 20 μg/mL	↑	Increased mesenchymal stem cell (MSC) migration in a dose-dependent manner.	[20]
OPN	1 μg/mL	↑	OPN reduced the number of organized actin cytoskeletons through the FAK and ERK pathways to increase BMSC migration.	[21]
OPN	1 μg/mL	↑	Reduced the number of organized actin cytoskeletons through the FAK and ERK pathways to increase BMSC migration.	[22]
OPN	1 μg/mL	↑	Cytoskeletal control of nuclear morphology and stiffness through the SUN1 proteins plays an important role in OPN-promoted BMSC migration.	[23]
OPN	1 μg/mL	↑	Chromatin organization was altered by the application of OPN via the ERK1/2 signaling pathway, which also contributed to BMSC migration.	[24]
Basic fibroblast growth factor (bFGF)	200 ng/mL	↑	Augmented the engraftment and differentiation capacity of transplanted BMSCs, recovering cardiac function.	[25]
bFGF	1 ng/mL up to 400 ng/mL	↓↑	Low concentrations led to an attraction of BMSCs, whereas higher concentrations resulted in repulsion.	[26]
Vascular endothelial growth factor (VEGF)-A	10 ng/mL	↑	Increased BMSC migration and proliferation.	[27]
Hepatocyte growth factor (HGF)	20 ng/mL	↑	Increased BMSC migration via PI3K pathways.	[28]
Insulin-like growth factor (IGF)-1	10 ng/mL	↑	Increased BMSC migratory responses via CXCR4 chemokine receptor signaling, which is PI3/Akt-dependent.	[29]
IGF-1	20 ng/mL	↑	Preconditioning of BMSCs with IGF-1 before infusion improved cell migration capacity and restored normal renal function after acute kidney injury.	[30]
Platelet-derived growth factor (PDGF)	50 ng/mL	↑	Increased BMSC migration significantly.	[31]
PDGF-B	40 ng/mL	↑	Increased recruitment/migration and differentiation of BMSCs.	[32]
Transforming growth factor (TGF)-β 1	100 pM	↑	Promoted the homing of BMSCs in myocardial ischemia/reperfusion injury and improved myocardial function.	[9]
TGF-β1	5 ng/mL	↑	Improved BMSC recruitment and wound closure in a syngeneic murine wound model.	[33]
TGF-β	1 ng/mL~100 ng/mL	↑	Activated noncanonical signaling molecules, such as Akt, ERK1/2, FAK, and p38, via TGF-β type I receptor to increase stem cell (BMSCs, BM-MSC-like ST2 cells) migration.	[34]

**Table 3:** Paracrine factors of transplanting BMSCs for tissue repair

Paracrine Factors	Animal Models	Outcomes	References
TGF-β, FGF-2, angiopoietin-2, VEGF-1	Rat myocardial infarction model	Triggered angiogenic and migratory effects at the site of the infarct to promote myocardial healing and improve the cardiac function.	[1]
NGF, HGF, IL-10, IL1-RA	NOD/SCID mouse model	Contributed to the prevention of apoptosis, increasing cell proliferation in the damaged liver.	[86]
TGF-β1, VEGF	Mouse burn injury model	Assisted in burn wound healing.	[2]
IGF-1	Mouse acute kidney injury model	Exerted beneficial effects on tubular cell repair in acute kidney injury.	[87]
Angiogenin, IL-8, MCP-1, and VEGF	Mouse hind limb ischemia model	Represented efficient biomarkers for predicting vascular regenerative efficacy of MSCs.	[4]
IGF-1, VEGF, EGF, and bFGF	Rat middle cerebral artery occlusion ischemia model	Induced functional improvement, reduced infarct volume, and showed neuroprotection in ischemic rats.	[88]
TGF-β	Rat stroke model	Suppressed immune propagation in the ischemic rat brain.	[89]
SDF-1, VEGF, HGF, and IL-6	Rat skin wound model	Enhanced the activity of dermal fibroblasts and keratinocytes to promote re-epithelialization and angiogenesis and, consequently, promoted wound healing.	[5]

**Table 4:** Conditioned medium from BMSCs for tissue repair

Conditioned Medium	Animal Models	Outcomes	References
IGF-1, VEGF, TGF-β1 and HGF	Rat periodontal defect model	Contributed to many processes of complicated periodontal tissue regeneration.	[3]
IL-6, IL-8	Rat hind limb ischemia model	Stimulated angiogenesis and tissue repair through an increase in homing of human cord blood-derived endothelial progenitor.	[90]
EGF, bFGF, PDGF, HGF, collagen type 1, and GDF-11	In vivo human test	Stimulated skin rejuvenation by increasing growth and ECM production.	[91]
Collagen types III and I and a high MMPs/TIMPs ratio	Mouse skin excisional wound model	Accelerated healing, with fewer scars compared with control groups.	[92]

## Abbreviations

AFSCs	Amniotic fluid-derived stem cells
bFGF	Basic fibroblast growth factor
BMSCs	Bone marrow-derived mesenchymal stem cells
BMSC-CM	Conditioned medium from BMSCs
CCL2	CC chemokine ligand 2
CCR2	CC chemokine receptor 2
CM	Conditioned medium
CRE	Cockroach allergen extract
CXCL7	Chemokine (C-X-C motif) ligand 7
CXCR4	CXC chemokine receptor 4
ECM	Extracellular matrix
EGF	Epithelial growth factor
ERK1/2	Extracellular signal-regulated kinase 1/2
ESCs	Embryonic stem cells
FAK	Focal adhesion kinase
Flt3	Fms-like tyrosine kinase 3
GDF-11	Growth differentiation factor-11
GFP	Green fluorescent protein
HGF	Hepatocyte growth factor
HSCs	Hematopoietic stem cells
HMGB1	High mobility group box 1
IGF-1	Insulin-like growth factor-1
IL1-RA	Interleukin-1 receptor antagonist
IL-6	Interleukin 6
IL-8	Interleukin-8
IL-10	Interleukin-10
JNK	c-Jun N-terminal kinase
LL-37	Leu-Leu-37
MAPK	Mitogen-activated protein kinase
MCP-1	Monocyte chemoattractant protein-1
MSCs	Mesenchymal stem cells
MTOC	Microtubule organizing center
NF-κB	Nuclear factor kappa B
NGF	Nerve growth factor
NOD/SCID	Non-obese diabetic/severe combined immunodeficiency
OPN	Osteopontin
PDGF	Platelet derived growth factor
PDGF-AB	Platelet derived growth factor isoforms AB
PDGF-BB	Platelet derived growth factor isoforms BB
PDGF-CC	Platelet derived growth factor isoforms CC
PDGFRs	Platelet-derived growth factor receptors
PDGFR-α	Platelet-derived growth factor receptor isoform α
PDGFR-β	Platelet-derived growth factor receptor isoform β
PI3K	Phosphatidylinositol 3-kinase
RAGE	Receptor for advanced glycation end
Rho A	Ras homolog gene family member A
SCF	Stem cell factor
SDF-1	Stromal derived factor-1
SUN1	Sad-1/UNC-84 1
TGF-β1	Transforming growth factor β1
TNF-α	Tumor necrosis factor
UC-MSC-CM	Umbilical cord blood-derived mesenchymal stem cells conditioned media
VEGF	Vascular endothelial growth factor
VEGFR	Vascular endothelial growth factor receptor
WJ-MSCs	Wharton’s jelly-derived mesenchymal stem cells
